# The influence of NH_4_ doping on the elastic properties of RbH_2_AsO_4_ crystals

**DOI:** 10.1038/s41598-025-27078-7

**Published:** 2025-11-07

**Authors:** B. Mroz, Z. Trybula, S. Mielcarek, A. Trzaskowska

**Affiliations:** 1 Faculty of Physics and Astronomy, Adam Mickiewicz University, Uniwersytetu Poznańskiego 2, Poznan, 61-614 Poland; 2 Institute of Molecular Physics, Polish Academy of Sciences, M. Smoluchowskiego 17, Poznan, 60-179 Poland

**Keywords:** Materials science, Physics

## Abstract

We report a systematic study of the elastic and dynamic properties of mixed Rb_1-x_(NH_4_)xH_2_AsO_4_ (RADA) crystals using high-resolution Brillouin light scattering over a broad temperature range (40–300 K). By varying the ammonium concentration, we reveal how $\:\text{N}{\text{H}}_{4}^{+}$ substitution alters the elastic stiffness tensor, phase transition temperatures, phonon damping, and order parameter relaxation dynamics. The results show a clear evolution from ferroelectric to antiferroelectric and proton-glass behavior as the ammonium content increases. We observe enhanced elastic softening, increased phonon scattering, and reduced relaxation times with doping, indicating a growing degree of structural disorder and frustration in the hydrogen-bond network. The observed changes in phonon thermal conductivity correlate with these elastic anomalies. Our findings highlight the potential of chemical substitution in tuning the mechanical and thermal properties of hydrogen-bonded ferroelectric crystals, with implications for designing adaptive low-temperature optical and memory materials.

**Supplementary Information:**

The online version contains supplementary material available at 10.1038/s41598-025-27078-7.

## Introduction

 Ferroelectric crystals are widely studied for their ability to reversibly switch between different ordered states in response to external stimuli such as electric fields, temperature, or light^1–3^. This property makes them ideal for tunable applications in sensing, switching, and memory technologies^4,5^.

In recent years, research on novel piezoelectric materials has advanced rapidly, particularly in the areas of hybrid perovskite ferroelectrics and molecular crystals. These materials, owing to their flexibility, low acoustic impedance, biocompatibility, and biodegradability, open new opportunities for applications in modern electronics, optoelectronics, and biomedical technologies. The discovery of large piezoelectric responses in lead-free hybrid perovskites, as well as high piezoelectricity in molecular crystals such as 2,2,3,3,4,4-hexafluoropentane-1,5-diol (HFPD), demonstrates that diverse crystalline systems can combine unique mechanical and electrical properties^6^. In this context, investigating the coupling between proton dynamics and lattice strain in classical hydrogen-bonded ferroelectrics is important not only for understanding the fundamental mechanisms of phase transitions but also for guiding the design of new piezoelectric materials with tunable properties and strong potential for use in sensors, actuators, and medical devices^6,7^.

Hydrogen phosphates and hydrogen arsenates, with the general formulas MH_2_PO_4_ and MH_2_AsO_4_ (M = K, Rb, Cs), are known as KDP family compounds and are isostructural crystals with tetragonal symmetry. These crystals are typical ferroelectric materials characterized by hydrogen bonding and are widely used in optical devices, such as lasers, optical communication systems, and optical switches. Extensive research has been conducted on crystals in this family^8–10^. Building upon the well-established properties of KDP-type ferroelectrics, recent efforts have turned to mixed systems where competing ordering tendencies give rise to novel physical phenomena.

 The electrical properties of materials are increasingly important in modern technologies. As a result, recent studies have focused on mixed ferroelectric–antiferroelectric crystals, particularly those based on KDP-type structures^11–16^. In particular, mixed ferroelectric–antiferroelectric crystals such as Rb_1 − x_(NH_4_)_x_H_2_PO_4_ (RADP) and Rb_1 − x_(NH_4_)_x_H_2_AsO_4_ (RADA) have garnered interest due to the competition between different types of proton ordering. Such competition introduces frustration in the hydrogen-bonding network, giving rise to glass-like behavior in a broad range of compositions^17–19^. The properties of RADP mixed crystals have been extensively studied^20–26^. At low temperatures, the phase behavior of these materials depends strongly on the ammonium concentration, with observed transitions into ferroelectric, antiferroelectric, or proton-glass states^17–19^. The dipole glass state in such frustrated ferroelectrics is challenging to detect experimentally and to describe theoretically. Korynevskii and Solovyan developed a theoretical framework for the dielectric properties of mixed crystals, which provides a clear signature for identifying the dipole glass transition. According to this model, the competition between different interactions in a complex crystalline matrix leads to multiple local states with varying occupancy, corresponding to local minima of the free energy. The formation of the dipole glass phase is determined by pair correlation functions of nearest-neighbor particles, which capture both the intensity and multiplicity of interparticle correlations, and thus explain the emergence of metastable, polystate arrangements characteristic of the dipole glass^27–30^.

Theoretical studies by Matsushita and Matsubara^31^ and experimental work by Courtens^20–22^ have highlighted the importance of understanding the antiferroelectric tendencies around $\:\text{N}{\text{H}}_{4}^{+}\:$ions in explaining the glass-like ordering in mixed crystals. Our findings indicate that the dynamics of the $\:\text{N}{\text{H}}_{4}^{+}$ group play a critical role in these transitions. Among the KDP family members, the mixed crystal Rb_1−x_(NH_4_)xH_2_AsO_4_ (RADA) stands out due to its unique phase diagram, which delineates the transitions between paraelectric and ferroelectric phases at lower ammonium concentrations and paraelectric-antiferroelectric transitions at higher concentrations^32,33^.

Despite extensive studies on the dielectric, thermal, and structural properties of RADP and RADA crystals^32–45^, a significant gap remains in understanding their elastic properties and phase transitions from a mechanical perspective. This is a crucial shortcoming, as the interaction between structural order parameters (e.g., polarization) and elastic strain is fundamental to the phase behavior of these materials. While the structural and dielectric characteristics of these crystals are well documented, their elastic response and phonon dynamics—particularly their composition- and temperature-dependence—are still poorly understood and have seen limited exploration through Brillouin light scattering. It is still unknown how the elastic response and phonon relaxation processes in RADA crystals change with increasing ammonium content, especially regarding the nature of the underlying phase transitions.

Brillouin light scattering (BLS) provides a powerful, non-invasive method for probing elastic constants and acoustic phonon dynamics across temperature-dependent phase transitions^46^. Through precise measurement of phonon frequencies and linewidths, BLS enables the detection of subtle fluctuations such as phonon softening and order-parameter relaxation. Furthermore, it offers insight into electrostrictive coupling mechanisms and the emergence of long-range polarization fluctuations characteristic of glassy states^47,48^. In particular, the strong coupling between proton dynamics and lattice strain in RADA crystals makes them ideal candidates for investigation using Brillouin light scattering, which directly probes acoustic phonons and elastic anomalies with high spectral resolution^49,50^.

This work investigates how varying concentrations of ammonium ($\:\text{N}{\text{H}}_{4}^{+}$) affect the elastic properties and phase transitions in Rb_1-x_(NH_4_)xH_2_AsO_4_ crystals using high-resolution Brillouin light scattering. Special attention is given to the temperature-dependent behavior of the main elastic constants (*c*_*11*_, *c*_*22*_, *c*_*33*_), order parameter relaxation times, and phonon thermal conductivity across distinct phase regions. The [110] crystallographic direction is particularly emphasized, as symmetry-breaking and elastic softening effects are expected along this axis. By systematically analyzing the evolution of acoustic phonon behavior and mechanical stability, this work provides new insights into the elastic signatures of ferroelectric, antiferroelectric, and proton-glass states. Specifically, we test the hypothesis that the substitution of rubidium ions by ammonium ions induces composition-dependent changes in the elastic stiffness tensor and phonon relaxation dynamics. These findings shed light on the coupling between elastic and polarization dynamics in hydrogen-bonded ferroelectrics.

## Results

### Elastic properties at room temperature

Brillouin light scattering spectra of phonons propagating along the main crystallographic directions [100], [010], [001], and [110] were collected using both 180-degree and 90 N geometries. The bulk phonon velocity υ was calculated using the equation:

1 $$\:\upsilon\:=\frac{{\lambda\:}_{0}v}{\sqrt{{n}_{i}^{2}+{n}_{s}^{2}-{2n}_{i}{n}_{s}\text{c}\text{o}\text{s}\left(\beta\:\right)}}$$

where λ_0_ is the wavelength of the laser, ν is the frequency shift determined from the Brillouin scattering spectra, *n*_*i*_ and *n*_*s*_ are the refractive indices along the incident (*i*) and scattered (*s*) light directions, and *β* is the scattering angle^50,51^.

Individual components of the elastic properties tensor *c*_*ijkl*_ were calculated based on the Christoffel Eqs^49,51^. :

2 $$\:\left|{c}_{ijkl}{q}_{j}{q}_{k}-\rho\:{\upsilon\:}^{2}{\delta\:}_{il}\right|=0$$

where *q*_*j*_ and *q*_*k*_ are the direction cosines, *ρ* is the crystal density, and *c*_*ijkl*_ is the elastic tensor. The methodology for calculating the refractive index is described in the Supplementary Materials.

**Fig. 1:**
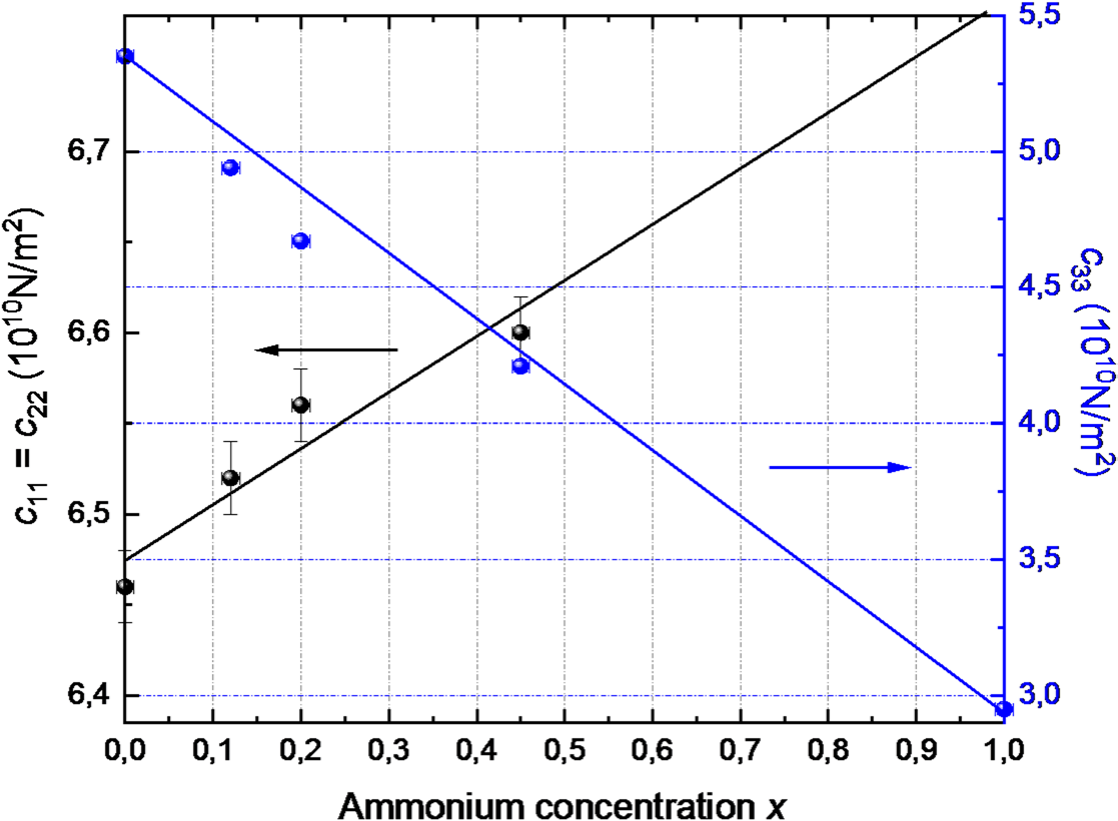
Variation of selected components of the elastic stiffness tensor (*c*_*11*_, *c*_*22*_, *c*_*33*_) at room temperature as a function of ammonium concentration (*x*) in RDA–ADA crystals.

The variation of the main components of the elastic properties tensor with ammonium concentration at room temperature is presented in Fig. 1. At room temperature, the values of *c*_*11*_ *= c*_*22*_ increase with ammonium concentrations, while for the RADA crystal with *x* = 0.45, the calculated *c*_*11*_ value is notably lower than expected, which indicates a different type of structural order. As the ammonium concentration increases, the *c*_*33*_ component of the elastic properties tensor decreases at room temperature. For the NH_4_H_2_AsO_4_ (ADA crystal) (*x* = 1), the value of *c*_*33*_ is approximately 3·10^10^ N/m^2^ (Fig. 1). This trend can be well approximated by a linear fit of the *c*_*33*_ component as a function of ammonium concentration, from *x* = 0 (RDA crystal) to *x* = 1 (ADA crystal). In contrast, no such trend is observed for *c*_*11*_ *= c*_*22*_, as in the ADA crystal, these values are constant and equal to *c*_*11*_ *= c*_*22*_ *=* 6.29 ·10^10^ N/m^2^^52^. The uncertainty of the *c*_*ij*_ values is approximately 0.02 ·10^10^ N/m^2^, the uncertainty for ammonium concentration is 0.01.

### Elastic softening as a function of temperature

**Fig. 2:**
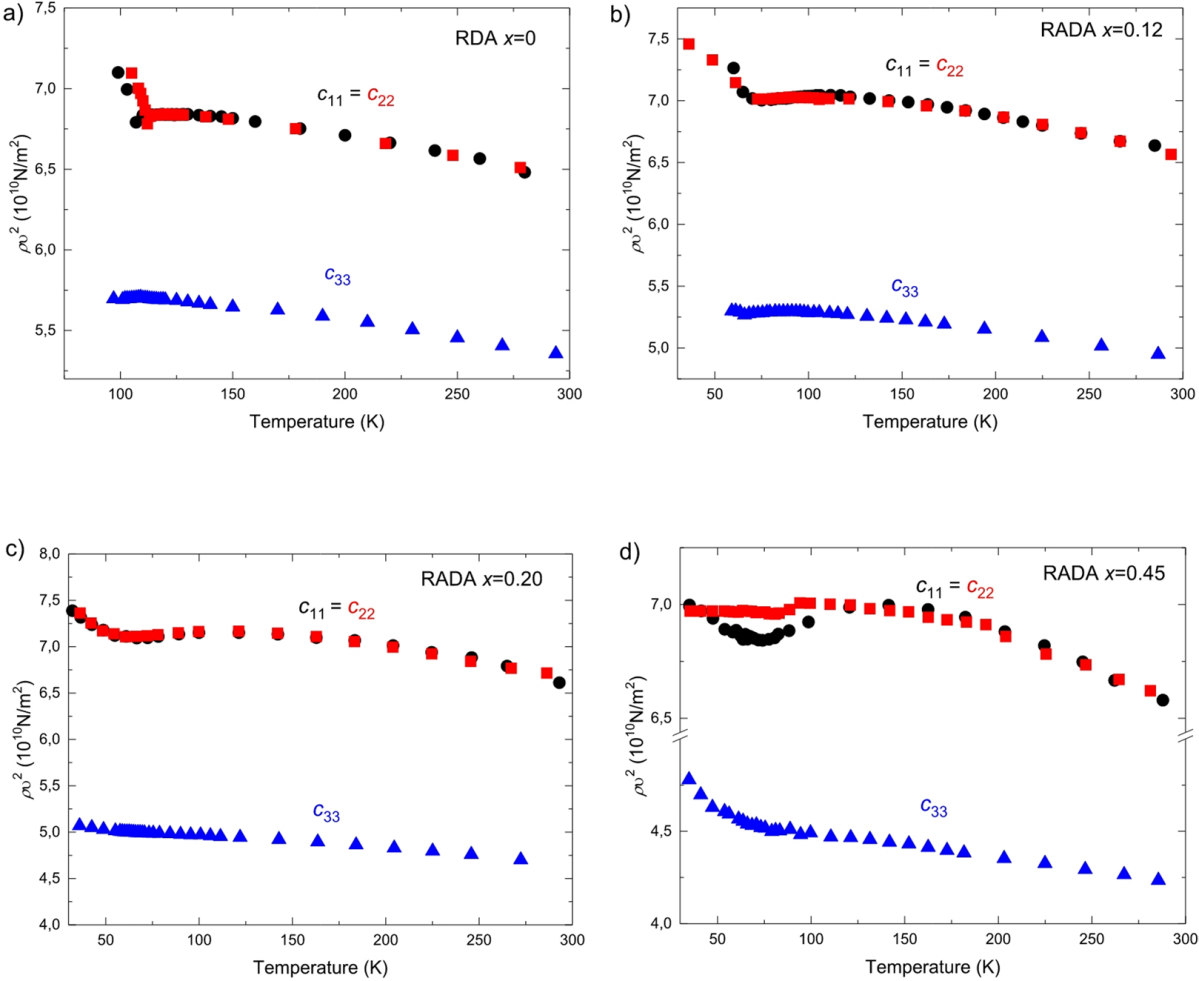
Temperature dependence of the elastic tensor coefficients *c*_*11*_, *c*_*22*_, and *c*_*33*_ for RDA and RADA crystals with varying ammonium concentrations (*x*). Values were extracted from Brillouin scattering spectra of longitudinal phonons propagating along the principal crystallographic directions. (Black circles - *c*_*11*_, red squares - *c*_*22*_, blue triangles - *c*_*33*_).

Knowledge of the refractive indices allowed the determination of the *c*_*11*_, *c*_*22*_, and *c*_*33*_ components of the elastic tensor as a function of temperature (Fig. 2). The uncertainty in the *c*_*ii*_ values is approximately 0.02·10^10^ N/m^2^. The size of the experimental points in the figure is larger than the corresponding uncertainty, and these uncertainties are already included in the points shown.

Temperature-dependent measurements revealed that all investigated crystals exhibit an initial increase in elastic tensor components upon cooling, followed by a decrease near the phase transition temperature (Fig. 2). For the RDA crystal, the minimum of the elastic tensor coefficient observed in Fig. 2 corresponds to the phase transition temperature^53^.

In the RADA *x* = 0.12 crystal, the minimum of the elastic components occurs at T_C_ = 76 K, indicating a transition to a mixed ferroelectric-paraelectric state^20^. For the RADA crystal with *x* = 0.20, the minimum in *c*_*11*_ *= c*_*22*_ occurs at T_C_ = 65 K. This temperature marks the onset of proton freezing in the crystal^33^. For the crystal with *x* = 0.45, the components of the elastic properties tensor reach a minimum at around 78 K.

The temperature dependence of the *c*_*33*_ component shows that anomalies near phase transitions are considerably less pronounced compared to those in *c*_*11*_ and *c*_*22*_. Similar effects have been observed in dielectric studies of RADA crystals^38^ and RADP crystals^22,26^. The largest differences are noted for the RADA crystal with *x* = 0.45, where the observed anomaly follows a different pattern compared to the other crystals.

### Phonon propagation along the [110] direction

**Fig. 3:**
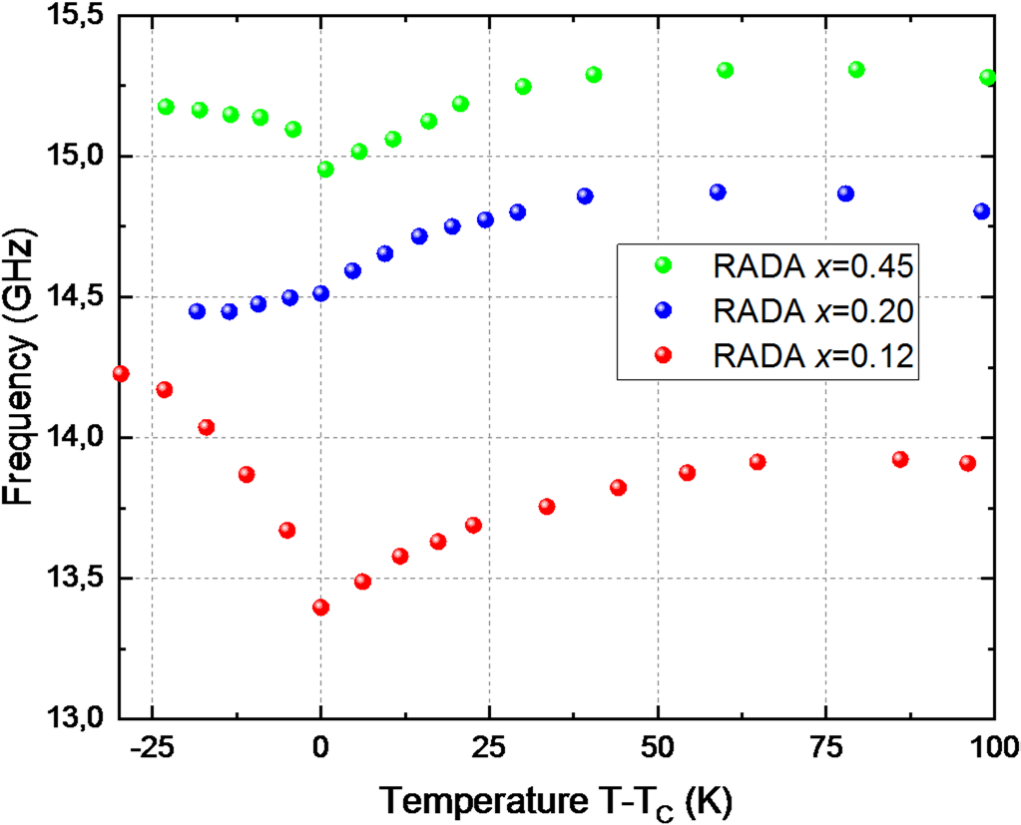
Temperature dependence of the longitudinal phonon frequency in the [110] direction for RADA crystals with varying ammonium concentration (*x*). The temperature is referenced to the transition point T_C_, which is determined from phonon softening behavior.

The phase transition occurring in the RDA crystal is classified as a ferroelectric and ferroelastic transition^46^. These transitions are usually marked by phonon softening phenomena. For the $\:\stackrel{-}{4}2m\:\to\:\:mm2$ phase transition, critical behavior of strain may be expected for the *e₆* order parameter component. This shear strain component also affects the propagation of longitudinal phonons traveling along the [110] direction. The results of the Brillouin scattering measurements of the longitudinal phonon mode propagating in the [110] direction are presented in Fig. 3. The uncertainty of the frequency values is approximately 0.04 GHz and is not visible in the figure, as it is smaller than the size of the plotted points. The plotted points already include these uncertainties.

The longitudinal phonon propagating in the [110] direction in the RDA crystal exhibits softening in the phase transition region (Fig. 3). The change in the phonon frequency is on the order of 0.5 GHz, or approximately 4%.

The dependencies shown in Fig. 3 suggest a strong indication of the influence of ammonium doping on the phonon propagation frequency in the [110] direction. As the concentration of the dopant increases, the phonon frequency exhibits a noticeable rise. This trend suggests that the ammonium atoms play a significant role in altering the phonon dispersion, likely due to their interaction with the crystal lattice and the modification of the force constants governing the vibrational modes.

**Fig. 4:**
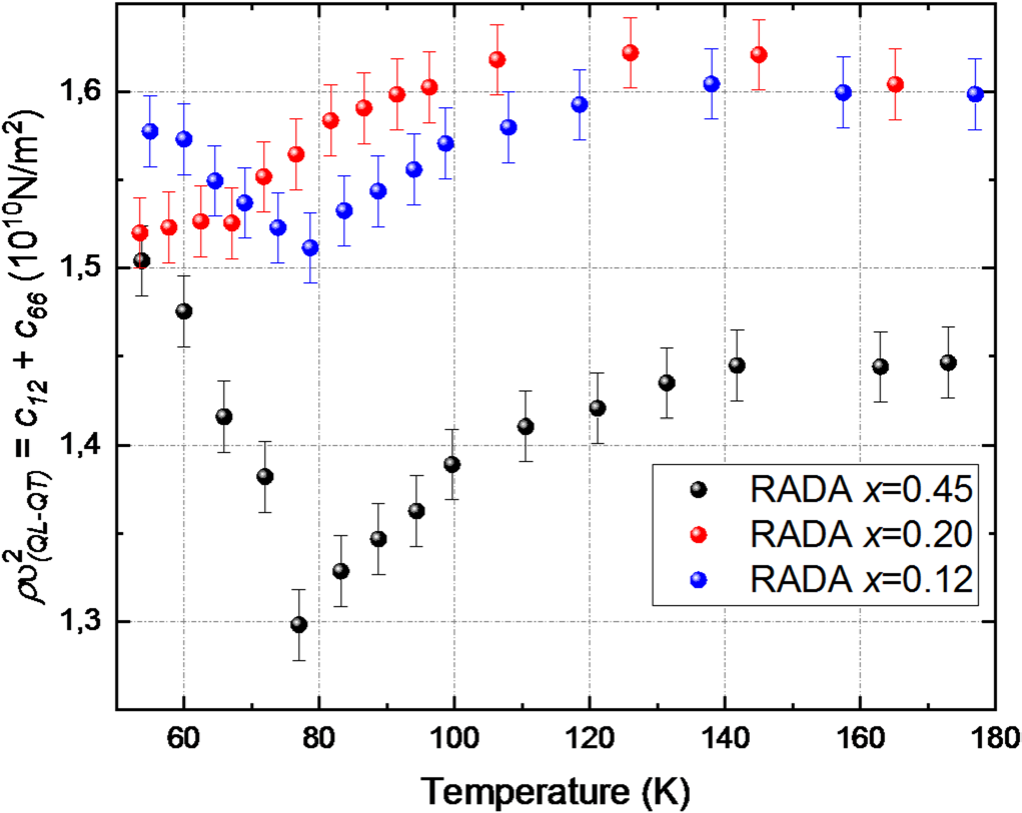
Temperature dependence of the elastic constant combination $\:\rho\:{\upsilon\:}_{(QL-QT)}^{2}$*= c*_*12*_ *+ c*_*66*_ for RADA crystals with different ammonium concentrations.

The longitudinal phonon propagating in the [110] direction can be described by the Christoffel Eq. (2)^49^ as a combination of the elastic tensor components: $\:\frac{1}{2}$ (*c*_*11*_ + *c*_*12*_ + 2 *c*_*66*_) = $\:\rho\:{\upsilon\:}_{QL}^{2}$. Additionally, the behavior of the transverse phonon propagating in the [110] direction was observed, which is described by the relation: $\:\frac{1}{2}$ (*c*_*11*_ – *c*_*12*_) = $\:\rho\:{\upsilon\:}_{QT}^{2}$. Consequently, the comparison of the obtained values of *ρυ*^*2*^ allowed for the determination of the combination of components $\:\rho\:{\upsilon\:}_{(QL-QT)}^{2}$ = *c*_*12*_ + *c*_*66*_ of the elastic tensor. The temperature at which *ρυ*^*2*^ reaches its minimum was determined based on the behavior of the mixed component $\:\rho\:{\upsilon\:}_{(QL-QT)}^{2}$ = *c*_*12*_ + *c*_*66*_ (Fig. 4). The uncertainty of the *ρυ*^*2*^ values is approximately 0.02 ·10^10^ N/m^2^. These temperatures are summarized in Table 1. It can be noted that the softening effect of the mode is most pronounced for the RDA crystal. An increase in ammonium concentration results in a smaller change in $\:\rho\:{\upsilon\:}_{QT}^{2}$within the phase transition temperature range.

**Table 1:** Transition temperatures corresponding to minima in $\:\rho\:{\upsilon\:}_{(QL-QT)}^{2}$ = *c*_*12*_ + *c*_*66*_ for RDA and RADA crystals.

Crystal	T (K)
RDA x = 0	105 ± 0.5
RADA *x* = 0.12	78 ± 0.5
RADA x = 0.20	66 ± 0.5
RADA *x* = 0.45	76 ± 0.5

Starting with the crystal RbH_2_AsO_4_ (RDA), which exhibits a ferroelectric phase transition at 105 K for the studied ammonium concentration, a shift in the transition temperature of more than 30 degrees was observed. This significant shift in the phase transition temperature suggests substantial changes in the nature of the transition.

## Discussion

The Brillouin light scattering results reveal clear composition- and temperature-dependent anomalies in the elastic stiffness tensor, which provide direct evidence of how $\:\text{N}{\text{H}}_{4}^{+}$ substitution modifies the structural symmetry and phase stability of RADA crystals.

For *x* = 0 and *x* = 0.12, a clear splitting between *c*_*11*_ and *c*_*22*_ occurs below the transition temperature, indicating lowered symmetry^20^. For *x* = 0.20, the anomalies in *c*_*11*_ and *c*_*22*_ are minimal, with a shallow minimum around 66 K. For *x* = 0.45, *c*_*11*_ ≠ *c*_*22*_ are in the range 100–48 K, consistent with antiferroelectric ordering, while their convergence at low temperatures (≈ 45 K) suggests a transition to a proton-glass state^39^. The decrease in *c*_*33*_ with increasing *x*, independent of phase type, supports preferential $\:\text{N}{\text{H}}_{4}^{+}$ orientation along the *a* and *b* axes, consistent with the crystallographic structure reported in^54^. A deeper analysis of *c*_*ij*_ has been included in the Supplementary Materials.

The present results confirm that increasing $\:\text{N}{\text{H}}_{4}^{+}$ concentration systematically lowers the elastic modulus *c*_*33*_, shifts the phase transition temperature to lower values, and enhances frustration within the hydrogen-bond network. This anisotropic evolution of the elastic response is consistent with previous dielectric studies^38^, but provides new mechanical evidence for symmetry lowering and frustration effects not accessible by purely electrical measurements.

Together, these results demonstrate that $\:\text{N}{\text{H}}_{4}^{+}$ substitution simultaneously weakens the lattice along the *c*-axis and enhances anisotropy within the *ab*-plane, establishing a direct structural origin of the observed competing ferroelectric, antiferroelectric, and glassy phases.

Similarly, for the *c*_*12 +*_ *c*_*66*_ components determined from phonons propagating along the [110] direction, it can be concluded that the ammonium concentration has a significant impact on phonon propagation, as evidenced by the shift of the phase transition temperature and the systematic changes in phonon frequency with increasing $\:\text{N}{\text{H}}_{4}^{+}$ content.

**Fig. 5:**
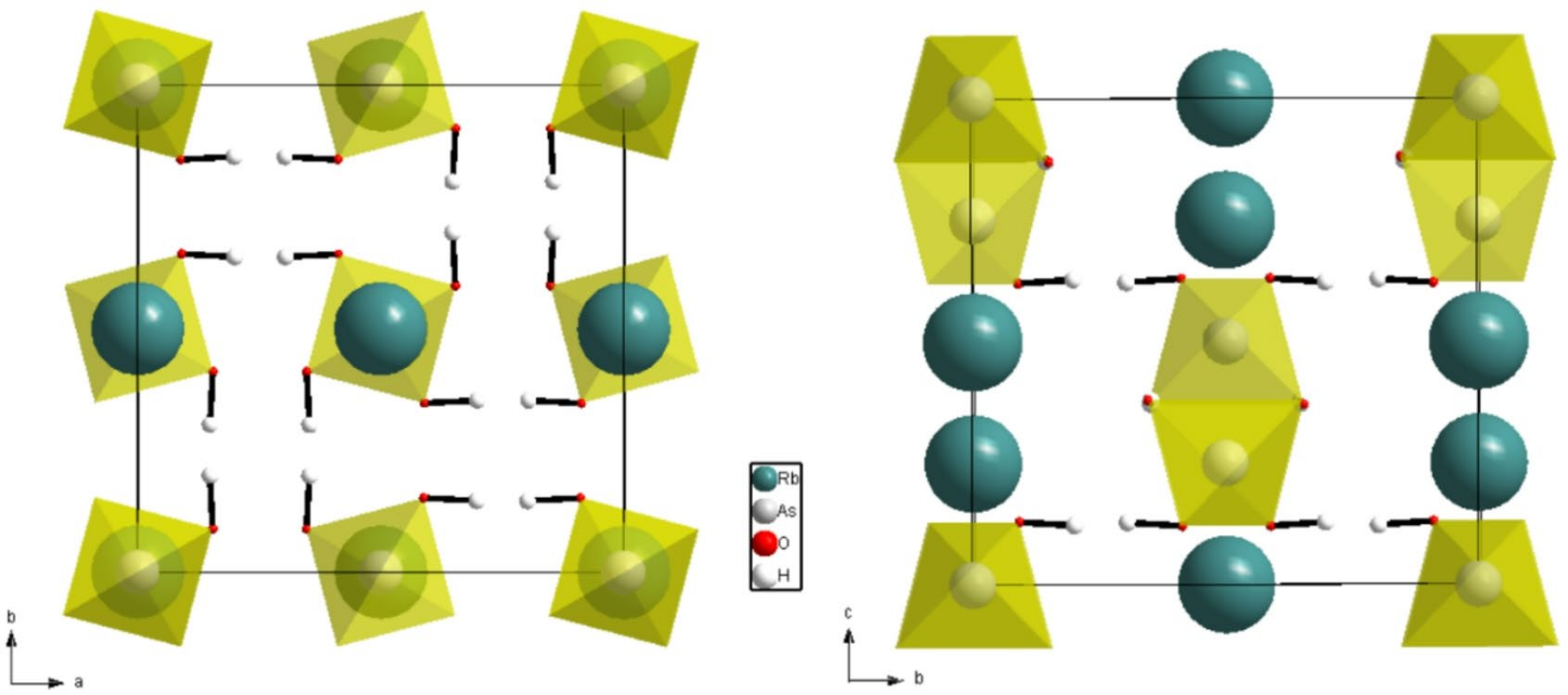
Crystallographic structure of the RDA crystal based on^54^.

On the microscopic level, $\:\text{N}{\text{H}}_{4}^{+}$ ions act as dynamic defects within the hydrogen-bond network. Their ability to undergo reorientational motions introduces local electric fields and strain fluctuations, which strongly couple to acoustic phonons. Considering the crystallographic structure of the RDA crystal (Fig. 5), it is important to analyze the specific occupancy sites of the ammonium ions in relation to the individual components of the elastic stiffness tensor. The observed increase in the elastic constants associated with longitudinal vibrations along the [100] and [010] directions, combined with the decrease in the *c*_*33*_ component corresponding to vibrations along the [001] direction, as a function of increasing ammonium concentration, suggests that the $\:\text{N}{\text{H}}_{4}^{+}$ ions preferentially align along the *a* and *b* axes. This preferential orientation may underlie the anisotropic elastic behavior observed in the studied mixed crystals.

To link the observed anisotropic elastic behavior with the dynamic effects of $\:\text{N}{\text{H}}_{4}^{+}$ ions, relaxation times obtained from Brillouin scattering provide a direct measure of the order-parameter dynamics. These times, derived from detailed analysis of the Brillouin spectra, reveal how local reorientational motions and strain fluctuations influence phonon propagation and elastic anomalies, as evidenced by temperature-dependent changes in the elastic constants. The single relaxation time τ can be found using the following relation^55,56^:

3 $$\:\frac{1}{2\pi\:\tau\:}=\frac{{\nu\:}_{\infty\:}^{2}-{\nu\:}_{0}^{2}}{{\varGamma\:}_{0}-{\varGamma\:}_{\infty\:}}$$

where *ν*_∞_ is the observed Brillouin shift, *ν*_0_ is the Brillouin shift not affected by the phase transition, Γ_∞_ is the observed Brillouin linewidth, and Γ_0_ is the Brillouin linewidth not affected by the phase transition.

The Brillouin linewidth observed far above the phase transition temperature was taken as Γ_0_. To determine the value of *ν*_0_, experimental data from temperatures ranging from approximately 150 K to room temperature were fitted with a linear function, which is attributed solely to lattice anharmonicity^55,56^. The values of Γ_0_ and *ν*_0_ for the individual studied crystals are summarized in Table 2.

**Table 2:** Values of Γ_0_ and *ν*_0_ were obtained for the studied crystals for the longitudinal phonon propagating in the [110] direction.

Crystal	Γ_0_ (GHz)	ν_0_ (GHz)
RDA *x* = 0	0.36 ± 0.02	13.69 ± 0.04
RADA *x* = 0.12	0.51 ± 0.02	14.00 ± 0.04
RADA *x* = 0.20	0.46 ± 0.02	14.86 ± 0.04
RADA *x* = 0.45	0.75 ± 0.03	15.44 ± 0.04

**Fig. 6:**
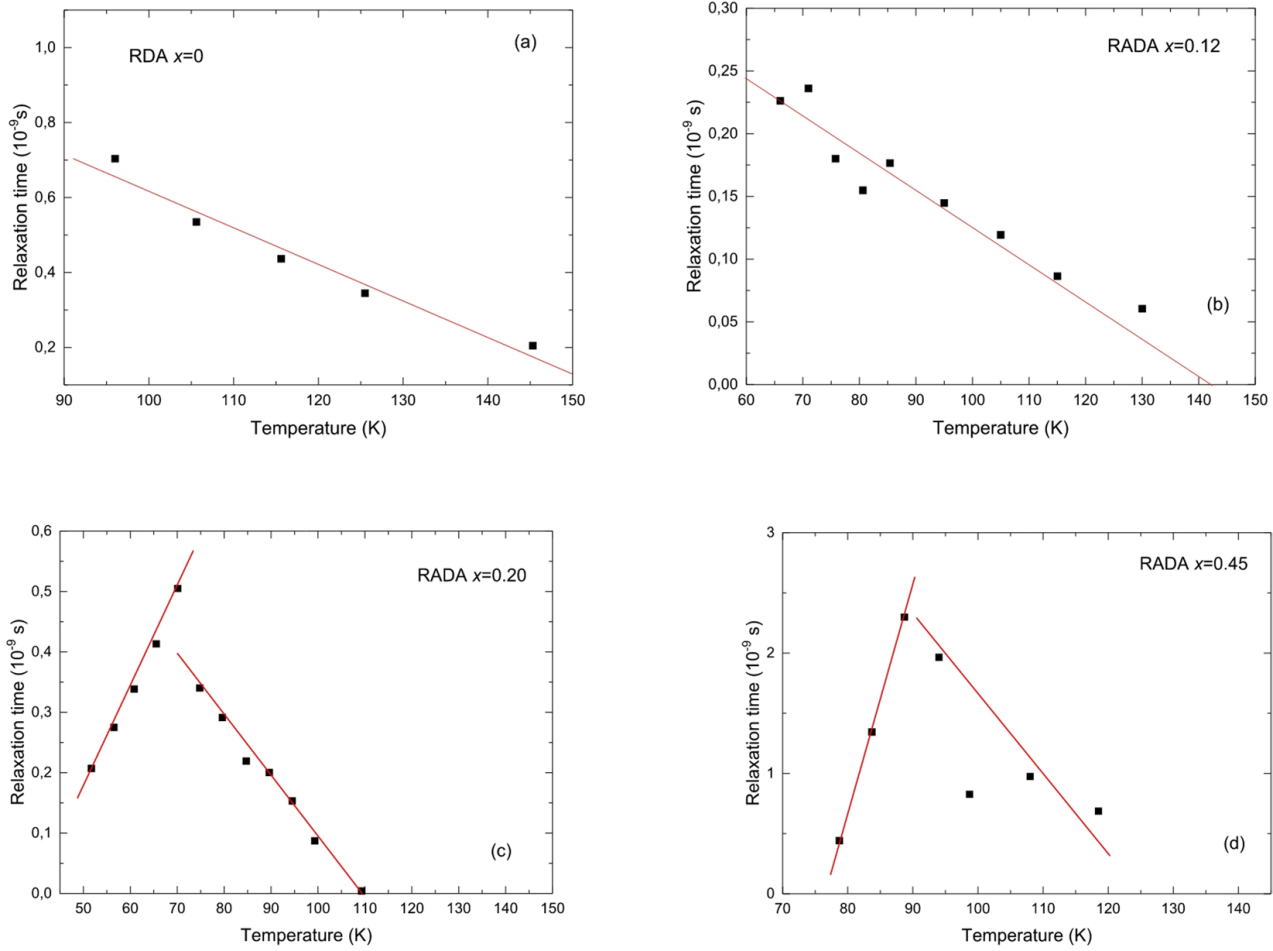
Temperature dependence of relaxation time for longitudinal phonons propagating in the [110] direction for RDA (*x* = 0 (a)) and RADA (*x* = 0.12 (b), *x* = 0.20 (c), *x* = 0.45 (d)) mixed crystals.

The temperature dependence of relaxation times for [110] longitudinal phonons is shown in Fig. 6 for all studied crystals. The uncertainty in determining the relaxation times arises from the inaccuracy of phonon frequency measurements and the determination of the full width at half maximum (FWHM) of the peaks. It is estimated to be approximately 0.03 ps.

Brillouin light scattering measurements reveal that all studied crystals exhibit fast relaxation dynamics. These relaxation processes occur on the picosecond timescale, characteristic of phonon dynamics in hydrogen-bonded crystals^57,58^. Despite this general behavior, the temperature dependence of the relaxation time τ varies significantly between compositions.

To gain deeper insight into these dynamics, we analyzed the data within the framework of the Landau–Khalatnikov (L–K) theory, which describes the critical slowing down of the order parameter near second-order phase transitions^59^. To better understand the relaxation dynamics near the transition, we compared our data with the Landau–Khalatnikov model, which describes critical slowing down of the order parameter near second-order phase transitions. According to this theory, the relaxation time diverges as:

4 $$\:\tau\:\left(T\right)\propto\:\frac{1}{\left|T-{T}_{C}\right|}$$

The RDA crystal undergoes a ferroelectric phase transition^33,34,53^, so the temperature dependence of the relaxation times approximates a linear function, consistent with Landau’s theory of phase transitions. Based on the dependence:

5 $$\frac{1}{\tau }\left( {T,q} \right)=\left| {\frac{{T - {T_C}}}{{{T_C}}}} \right| \cdot \frac{1}{{{\tau _1}}}+\frac{1}{{{\tau _2}}}\left( q \right)$$

The relaxation times *τ*_1_ and *τ*_2_ can be determined. Here, *τ*_1_ represents the temperature-dependent, critical relaxation time associated directly with the phase-transition dynamics (e.g., soft-mode behavior or dipole ordering), while *τ*_2_ corresponds to a relatively constant relaxation time arising from other mechanisms (such as diffusive processes, mechanical damping, etc.) that are present regardless of temperature^60^. The best fit to the experimental data using Eq. (7) yields *τ*_1_ = 3,4±0,5 ps and *τ*_2_ = 21,1±3,9 ps. It should be noted that the relaxation time *τ*_1_ is longer than typically observed for order-disorder phase transitions^59,60^. However, the values of *τ*_1_ suggest that the phase transition involves both order-disorder and displacement types^59^. The obtained values of *τ*_1_ are interpreted as fluctuations accompanied by a volume change at the phonon frequency.

The crystal RADA *x* = 0.12 also exhibits a linear dependence of relaxation times on temperature, which further confirms the ferroelectric nature of the phase transition. We calculated the relaxation times from the fit according to Eq. (7) as follows: *τ*_1_ = 7,7±1,0 ps and *τ*_2_ = 18,8±3,0 ps. Considering that the crystal with concentration *x* = 0.12 does not exhibit a pure ferroelectric transition but rather transitions to a mixed ferroelectric-paraelectric state, these large relaxation times can be attributed to this mixed phase behavior.

The relaxation times exhibit distinctly different behavior for the crystal RADA *x* = 0.20, with a noticeable change in the relaxation behavior of the order parameter at approximately 70 K. This confirms previously observed anomalies in the frequency and elastic components of this crystal. Simultaneously, a markedly different relaxation time behavior as a function of temperature is observed, indicating a different type of phase transition.

For the crystal RADA *x* = 0.45, the relaxation time shows an anomaly at around 90 K. This crystal, with the given concentration, is challenging to categorize into a specific phase transition group. On one hand, the temperature of the elastic anomaly, approximately 76 K, suggests considering the onset of a freezing process associated with a glassy transition. On the other hand, the temperature of the relaxation time anomaly, around 90 K, indicates an antiferroelectric transition in this crystal^33,34^. This analysis of relaxation times is based on the softening of the elastic tensor components, specifically for vibrations propagating in the [110] direction.

The Landau–Khalatnikov approach assumes a continuous second-order phase transition and a single relaxation channel. In the case of RADA, deviations from this behavior, particularly for *x* = 0.20 and 0.45, suggest that disorder-induced broadening of relaxation spectra or the coexistence of multiple relaxation mechanisms may play a role. This highlights the need to consider models beyond the simple Landau–Khalatnikov framework, such as Vogel–Füller^22^ approaches commonly applied to proton-glass systems.

To better understand the nature of these phase transitions and the mechanisms underlying the observed relaxation behavior, it is essential to consider the energetic aspects of phonon dynamics, particularly the activation energy involved in phonon-mediated processes.

According to the literature, phonon activation energy in crystalline materials generally represents the energy needed for phonon-assisted processes such as thermal relaxation, scattering, or defect-mediated diffusion, and typically lies within the range of several tens to a few hundred meV, depending on the phonon branch and the dominant scattering mechanism^61–63^. For example, the activation energy for acoustic phonon damping can reach values up to about 0.1 eV in the low-temperature region due to strong phonon–phonon and phonon–defect interactions^64^. In the studied RADA crystals, the estimated activation energies (Table 3) indicate that the dynamical behavior is influenced by such interactions and correlate with the characteristic phonon relaxation times derived from Brillouin scattering. The increasing phonon damping observed with $\:\text{N}{\text{H}}_{4}^{+}$ doping suggests stronger phonon-defect scattering due to local structural disorder and reorientational dynamics of ammonium ions.

The use of the equations:

6 $$\rm In \tau = In \tau_1 + \textit{K}_\textit{B}E_a \cdot T$$

7 $$\:{\kappa\:}_{ph}\approx\:\frac{1}{3}{C}_{v}{v}^{2}\tau\:$$

enables the calculation of the phonon activation energy (E_a_), while T is temperature, *k*_B_ is the Boltzmann constant, and τ is the relaxation time and phonon thermal conductivity where *C*_*v*_ is volumetric specific heat, *v* – velocity of phonons. The result obtained from BLS provides a local or partial estimation of the contribution of long-wavelength acoustic phonons to the thermal conductivity (κ_ph_).The corresponding data are presented in Table 3.

The relaxation times determine the mean free path of phonons and directly affect the phonon thermal conductivity, which quantifies the ability of phonons to transport heat through the crystal lattice without excessive scattering. The strong increase in phonon linewidth near the transition temperature is consistent with critical slowing down, as described by Landau–Khalatnikov-type relaxation of the order parameter. As the ammonium concentration increases, stronger disorder and enhanced anharmonicity lead to shorter relaxation times and higher phonon scattering rates, which in turn reduce the thermal conductivity. Understanding this interplay between activation energy, relaxation dynamics, and thermal conductivity is crucial for controlling heat transport and optimizing functional properties in hydrogen-bonded ferroelectrics, particularly in materials designed for advanced optical and low-temperature memory applications. The activation energies and phonon thermal conductivities for the studied crystals are summarized in Table 3.

**Table 3:** Activation energy and phonon thermal conductivity for studied materials at RT.

	RDA (x = 0)	RADA (x = 0.12)	RADA (x = 0.20)	RADA (x = 0.45)
Activation energy (meV)	56.7 ± 0.3	30 ± 0.2	16.7 ± 0.1	29.6 ± 0.2
Phonon thermal conductivity (W/mK)	25.3 ± 0.1	24.8 ± 0.1	23.6 ± 0.1	20.6 ± 0.1

The estimated activation energies indicate that the dynamical processes are mainly of an order–disorder type processes governing the phase transitions in RDA and $\:\text{N}{\text{H}}_{4}^{+}$ - substituted RADA crystals are mainly of an order–disorder type. The relatively low activation energies, typically within the range of tens of meV, are consistent with the thermal reorientation of hydrogen bonds and local proton hopping mechanisms that dominate the relaxation dynamics in these hydrogen-bonded ferroelectrics.

The calculated phonon thermal conductivity reflects the combined effects of the lattice stiffness and the phonon scattering rates. This behavior highlights the interplay between chemical substitution and anharmonic phonon interactions, which can be effectively tuned to control heat transport properties in RADA-type crystals.

Let us analyze the above results from the perspective of group theory. The RADA crystals undergo a phase transition that is both ferroelectric and ferroelastic. In the case of the 4̅2m → mm2 phase transition, the critical behavior of strain is expected for the *e*_*6*_ component. According to Landau theory, a phase transition induced by the representation Γ may be continuous only if the symmetric product [Γ]³ does not contain the identity representation.

The Lifshitz condition, based on spatial homogeneity arguments of the low-symmetry phase, states that if the antisymmetric square [Γ]² is contained in the vector representation V, then a linear invariant in spatial derivatives of the order parameter can be constructed.

In our case, as a variable transforming according to this one-dimensional representation, we may choose either the polarization P_z_ or the shear strain *e*_*12*_ as the relevant variable. The prototype 4̅2m tetragonal phase (space group D_2d_) becomes unstable against a B₂ distortion and undergoes a transition to the mm2 polar orthorhombic phase (space group C_2V_). Since both *z* and *xy* transform according to the B₂ irreducible representation of 4̅2m, a polar optic soft mode (P_z_) and a transverse acoustic soft mode (*e₆*), which are linearly coupled, both condense at the transition. The linear coupling coefficient in this case is the piezoelectric constant *d₃₆*. This coupling between the soft optic and transverse acoustic modes leads to optic–acoustic hybridization, resulting in the softening of the elastic constant near T_C_. Such behavior is well known for KDP-type crystals, where the elastic constant *c*_*66*_ tends to zero at T_C_. However, in RADA crystals, the acoustic modes are not completely soft, and more than one vibration exhibits partial softening in the vicinity of T_C_. Therefore, the question of which parameter—polarization or strain—should be regarded as the *primary order parameter* remains open.

These findings provide a deeper insight into the microscopic mechanisms that govern the thermal and elastic behavior of mixed RADA crystals and confirm the potential of $\:\text{N}{\text{H}}_{4}^{+}$ doping as a tool for tailoring both phase transition characteristics and phonon-mediated thermal transport.

This clear correlation between $\:\text{N}{\text{H}}_{4}^{+}$ doping, elastic anomalies, and phonon dynamics demonstrates the tunability of both mechanical and thermal responses in RADA crystals. The results reveal that these systems are highly sensitive to ammonium content, reflecting a delicate balance between ferroelectric, antiferroelectric, and glass-like interactions. By enhancing lattice disorder and modifying phonon scattering, $\:\text{N}{\text{H}}_{4}^{+}$ substitution effectively reduces thermal conductivity, offering a route to engineer materials with tailored heat transport properties. Such control is particularly relevant for thermoelectric and phononic applications, where efficient thermal management is essential. Furthermore, the ability to stabilize different phase states—from ferroelectric through antiferroelectric to proton-glass—underscores the potential of RADA for adaptive optoelectronic components and low-temperature memory devices, in which both phase stability and proton dynamics can be functionally exploited.

## Conclusion

This study provides a comprehensive analysis of the elastic and dynamic properties of Rb_1-x_(NH_4_)xH_2_AsO_4_ crystals using high-resolution Brillouin light scattering. This study demonstrates increasing ammonium concentration systematically reduces the elastic stiffness—particularly the *c*_*33*_ modulus—enhances phonon damping, and shortens relaxation times. These effects are attributed to growing structural frustration and dynamic disorder introduced by $\:\text{N}{\text{H}}_{4}^{+}$ ions. The shift and broadening of phase transition regions, as well as the decrease in phonon thermal conductivity, highlight the strong interplay between lattice dynamics and the stability of ferroelectric, antiferroelectric, and proton-glass states. Brillouin spectroscopy thus proves to be a sensitive probe for detecting subtle mechanical signatures of complex phase behavior in hydrogen-bonded systems. The demonstrated tunability of elastic and thermal properties suggests new opportunities for tailoring advanced ferroelectric, thermoelectric, and phononic materials for low-temperature and adaptive device applications.

The main points:

- Increasing $\:\text{N}{\text{H}}_{4}^{+}$ content induces elastic softening and enhances phonon scattering.

- Phase transition temperatures shift and overlap with doping.

- Proton disorder critically affects both dynamic and thermal responses.

- Elastic anomalies can be effectively tracked via BLS measurements.

The conducted study provides important insights both for the development of modern technologies and for advancing the fundamental understanding of phase transitions in hydrogen-bonded crystals. The Rb_1-x_(NH_4_)ₓH_2_AsO_4_ system, owing to its sensitivity to $\:\text{N}{\text{H}}_{4}^{+}$ substitution, enables precise tuning of elastic and dynamic properties, thus opening avenues for the design of materials with controlled thermal conductivity and tailored dielectric response. The observed anomalies in elastic behavior and phonon damping lay the foundation for applications in low-temperature optical components, such as fiber-optic switches, light modulators, and adaptive ferroelectric memory devices. Simultaneously, the analysis of order parameter relaxation dynamics and the emergence of proton-glass states makes a significant contribution to the theoretical framework describing critical behavior in proton-ordered systems and their phase transitions. These findings illustrate how subtle structural modifications and frustration within the hydrogen-bond network can lead to the formation of complex, non-classical states of matter, bridging the fields of ferroelectricity, glass physics, and disorder theory.

## Method

### Crystals

The mixed crystals of Rb_1-x_(NH_4_)_x_H_2_AsO_4_ were synthesized via the slow evaporation method from an aqueous solution containing RbH_2_AsO_4_ (RDA), with a ferroelectric phase transition temperature T_C_ = 105 K, and NH_4_H_2_AsO_4_ (ADA), with an antiferroelectric phase transition temperature T_N_ = 216 K, combined in appropriate molar ratios^36,37,39^. In contrast to RADP mixed crystals, where the ferroelectric phase transition temperature of RbH_2_PO_4_ (T_C_) and the antiferroelectric phase transition temperature of NH_4_H_2_PO_4_ (T_N_) are nearly identical (T_C_ ≈ T_N_), RADA crystals exhibit a significant difference between the transition temperatures of RbH_2_AsO_4_ (T_C_) and NH_4_H_2_AsO_4_ (T_N_), where T_C_ < T_N_. The resulting RADA crystals, measuring 3 × 3 × 5 mm³, were transparent and exhibited high optical quality. These crystals crystallized in the tetragonal system, specifically in the point group $\:\stackrel{-}{4}2m$. Notably, the c-axis was the preferred direction of crystal growth.

The ammonium concentration x in the RADA crystals was varied, and determined through rubidium analysis using flame atomic absorption spectroscopy. The ammonium concentration of RADA crystals was: *x* = 0.12 ± 0.01, 0.20 ± 0.01, 0.45 ± 0.01, and for RDA crystal, the ammonium concentration was 0 (*x* = 0). For the RDA crystal, the ammonium concentration was confirmed to be 0 (*x* = 0). The RDA and RADA crystals belong to the KDP crystal family. The optical axis [001] (parallel to the tetragonal *c*-axis) is the fastest growth direction; therefore, the crystals often elongate along this axis. The typical morphology of RADA is a tetragonal bipyramid bounded by the (101) or (100) planes, with the (100) faces being relatively slow-growing and stable. Samples were prepared along the main crystallographic axes, with approximate dimensions of 2 × 2 × 3 mm³.

### Brillouin experiment

Brillouin spectra of the RADA crystals were studied using a six-pass tandem BLS (JRS Scientific Instruments), providing a contrast of 10^10^^65,66^. A single-mode Nd: YAG diode-pumped laser emitting a second harmonic at a wavelength of λ_0_= 532 nm with an output power of 200 mW (Excelsior, Spectra Physics) served as the light source. The Brillouin spectra give information about the frequency of studied phonons in a particular direction of propagation of the phonons. The uncertainties are 0.04 GHz for the frequency. Detailed information on the experimental setup can be found in Refs^65,66^.. Brillouin light scattering was observed in the temperature range from 300 K to 40 K, with a temperature stabilization of ± 0.1 K.

The elastic properties of a given medium, characterized by the elasticity tensor *c*_*ij*_, are intrinsically linked to the propagation velocity of acoustic waves within that medium. These properties were determined using the Christoffel Eqs^49,67,68^. At room temperature, RADA crystals are defined by six independent elastic constants: *c*_*11*_, *c*_*33*_, *c*_*44*_, *c*_*66*_, *c*_*12*_, and *c*_*13*_. However, it was not possible to determine the components of the elasticity tensor for RADA crystals in their low-temperature phase. According to the phase diagram, this phase is not a pure ferroelectric phase, as the samples exhibit characteristics of a glassy phase (proton glass state). Consequently, it is also not possible to assign a specific point group to the low-temperature phase. This complexity further complicates the assignment of particular components of the elasticity tensor to the phonons under investigation at low temperatures.

## Supplementary Information

Below is the link to the electronic supplementary material.

Supplementary Material 1

## Data Availability

The authors declare that the data supporting the findings of this study are available within the paper, its supplementary information files.
