# Supplementary material for: The influence of NH4 doping on the elastic properties of RbH2AsO4 crystals
Source: Sci Rep. 2025 Nov 7;15:39140. doi: 10.1038/s41598-025-27078-7 (PMC12595090; doi:10.1038/s41598-025-27078-7)
Supplement: Supplementary file 1 — Supplementary Material 1 [file 41598_2025_27078_MOESM1_ESM.docx]

**Supplementary materials to The Influence of NH_4_ Doping on the Elastic Properties of RbH_2_AsO_4_ Crystals**

B Mroz^1^, Z Trybula^2^, S Mielcarek^1^, A Trzaskowska^1*^

^1^ Faculty of Physics and Astronomy, Adam Mickiewicz University, Uniwersytetu Poznańskiego 2, 61-614 Poznan, Poland

1. Institute of Molecular Physics, Polish Academy of Sciences, M. Smoluchowskiego 17, 60-179 Poznan, Poland

*E mail: olatrzas@amu.edu.pl

1. **Index of refraction**

Brillouin light scattering spectra of phonons propagating along the crystallographic directions [100], [010], [001], and [110] were collected using both 180-degree and 90N geometries. For isotropic materials, such as cubic crystals, thin films, or polymers, additional scattering geometries-namely 90A and 90R-can be employed to determine the complete set of elastic constants and refractive indices [S1]. The application of these geometries enables the calculation of refractive index in a given medium according to the following relationships:

$n=\frac{\Delta\nu_{90N}}{\Delta\nu_{90A}}$ (1)

$n=\sqrt{\frac{1}{2}\left(\left(\frac{\Delta\nu_{90R}}{\Delta\nu_{90A}}\right)^{2}+1\right)}$ (2)

$n=\frac{\Delta\nu_{180}}{\Delta\nu_{90A}\sqrt{2}}$ (3)

where: *Δν* is the Brillouin shift of the acoustic phonon measured using a specific measurement geometry, and *n* is the refractive index of the given medium.

Although this method is primarily applied to isotropic systems, it can also be extended to anisotropic crystals under specific symmetry and propagation conditions. The investigated RDA (with ammonium concentration *x* = 0) and RADA crystals in the high-temperature phase exhibit tetragonal symmetry, for which the [100] and [010] directions are equivalent. In the case of studying the longitudinal phonon propagating in the [110] direction using the 90N and 90A geometries, the only refractive index that needs to be considered is *n_x_ = n_y_*. This allows the crystal to be treated as isotropic in this specific case. Thus, it is possible to determine the refractive index *n_x_ = n_y_* of the studied materials by comparing the frequencies of longitudinal phonons propagating in the [110] direction according to equations (1–3). To determine the individual refractive indices characteristic of RADA crystals, measurements of the longitudinal phonon propagating in the [110] direction of the studied crystals were performed using the 90A geometry. This procedure enables the determination of the refractive index from the measured phonon frequencies. These values are summarized in Table S1.

Table S1. Refractive index values for the Rb_1-x_(NH_4_)_x_H_2_AsO_4_ crystals.

|  | RDA | RADA (*x*=0.12) | RADA (*x*=0.20) | RADA (*x*=0.45) |
| --- | --- | --- | --- | --- |
| *n*_x_ | 1.48 | 1.51 | 1.52 | 1.53 |
| *n*_z_ | 1.45 | 1.47 | 1.47 | 1.48 |

1. **Debye temperature**

Another parameter that can be determined from Brillouin light scattering studies is the Debye temperature. The Debye temperature reflects the overall stiffness of the crystal lattice and the intensity of acoustic phonon vibrations. Its variations indicate changes in the elastic, thermal, and dynamic properties of the material - all of which are critical for its technological applications. The Debye temperatures for the studied crystals were determined based on the following relationship:

 (4)

Where is the reduced Planck’s constant, *k_B_* – Boltzmann’s constant, *N*_V_ – number of atoms in a unit volume. The obtained results are presented in Table S2.

Table S2. The Debye temperatures Θ_D_ (K) for RADA crystals.

| Crystal | Θ_D_ (K) |
| --- | --- |
| RDA *x* = 0 | 437 |
| RADA *x* = 0.12 | 440 |
| RADA *x* = 0.20 | 447 |
| RADA *x* = 0.45 | 468 |

The Debye temperature increases with rising ammonium concentration, which is consistent with observations for Rb_1-x_(NH_4_)_x_H_2_AsO_4_ crystals [S2-S6]. In the context of mixed crystals such as Rb_1-x_(NH_4_)_x_H_2_AsO_4_, the observed decrease in Debye temperature with increasing $NH_{4}^{+}$concentration suggests lattice softening, enhanced structural disorder, and stronger phonon damping, which correlates with the transition from ferroelectric phases to a proton-glass state.

1. **Original data from Brillouin spectroscopy**

The original longitudinal phonon frequencies measured in the studied materials are presented in Figure S1

Figure S1. Frequencies of longitudinal phonons along the main crystallographic directions: (a) [100], (b) [010], (c) [001].

An example Brillouin spectrum obtained for one of the RADA crystals is shown in Figure S2.


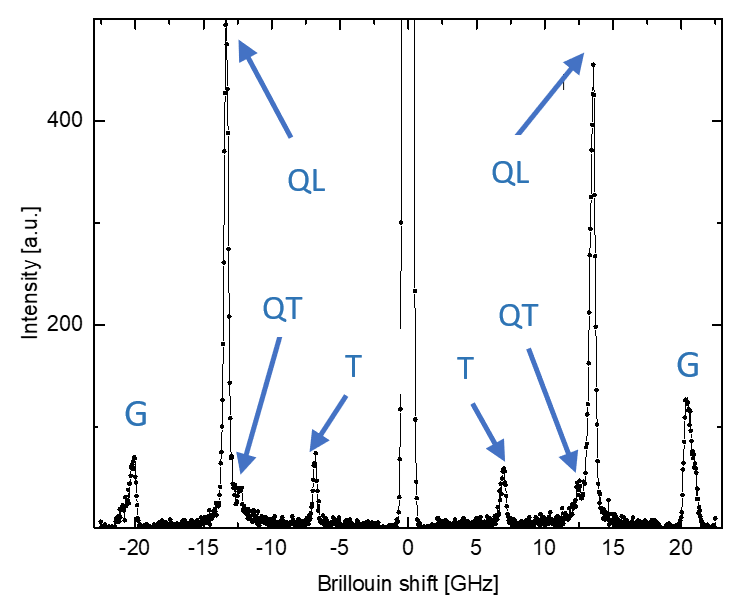


Figure S2. Typical Brillouin spectrum of RADA crystal measured along [110]. Ghost lines (G) determine the free spectral range (FSR) of the apparatus. In this case FSR = 20 GHz or $\frac{2}{3}$ cm^-1^_._ QL and QT state for quasi-longitudinal and quasi-transverse modes, respectively. T states for pure transverse mode.

1. **Discussion and analysis of *c_ij_***

To assess how acoustic phonons propagating along the principal crystallographic directions are affected by varying ammonium content, we examined the temperature dependence of the elastic stiffness tensor components *c_11_*, *c_22_*, and *c_33_* in selected compositions.

In the pure RDA crystal (*x* = 0), pronounced anomalies are observed in *c_11_*, *c_22_*, and *c_33_* around 105 K. Below the phase transition, the values of these components diverge, indicating a transition to a low-symmetry ferroelectric phase. A similar pattern is found in the RADA crystal with *x* = 0.12, where the anomaly in *c_11_* and *c_22_*, appears near 78 K. This temperature corresponds to the paraelectric–ferroelectric transition, as indicated by the established phase diagram for RADA systems [S2]. In the low-temperature phase, the anisotropy of *c_11_* and *c_22_* again reflects the loss of tetragonal symmetry.

For the RADA crystal with *x* = 0.20, the situation differs. The components *c_11_* and *c_22_* remain equal throughout the measured temperature range. The anomaly at ~66 K is weaker and appears as a shallow minimum, possibly associated with the onset of proton freezing rather than a conventional symmetry-breaking phase transition [S7].

The most complex behavior is observed in the RADA crystal with *x* = 0.45. Here, *c_11_* and *c_22_* are nearly equal at both high (T > 100 K) and low (T < 48 K) temperatures, but in the intermediate range (100 K > T > 48 K), they diverge. According to the phase diagram, this sample is likely to undergo a paraelectric–antiferroelectric transition near 90 K, followed by a transition into a proton-glass state below ~45 K. The restoration of the equality of *c_11_* and *c_22_* at low temperatures is consistent with the expected isotropy of the glassy phase [S8]. Interestingly, this composition is also the only one exhibiting an anomalous increase in the *c_33_* component upon cooling.

Across the studied series, an increase in room-temperature values of *c_11_* and *c_22_* is observed with rising ammonium content, while *c_33_* systematically decreases. The behavior of *c_33_* appears to be largely insensitive to the nature of the phase transition, suggesting that lattice rigidity along the c-axis is only weakly affected by proton ordering phenomena.

References

[S1] Kruger, J. K., Marx, A., Peetz, L., Roberts, R. & Unruh, H. G. Simultaneous determination of elastic and optical properties of polymers by high performance Brillouin spectroscopy using different scattering geometries. Colloid and Polymer Sci 264, 403-414 (1986).

[S2] Courtens, E. & Vacher, R. Spectroscopy of glassy systems with a relaxation-time distribution: Application to Brillouin scattering on Rb_0.65_(NH_4_)_0.35_H_2_PO_4_. Phys. Rev. B 35, 7271-7274 (1987).

[S3] Courtens, E., Vacher, R. & Dagorn, Y. Brillouin spectroscopy of polarization fluctuations in a Rb_1-x_(NH_4_)_x_H_2_PO_4_ glass. Phys. Rev. B 33, 7625-7636 (1986).

[S4] Courtens, E. Vogel-Fulcher Scaling of the Susceptibility in a Mixed-Crystal Proton. Phys. Rev. Lett. 52, 69-72 (1984).

[S5] Grimm, H., Courtens, E., Dorner, B. & Monkenbusch, M. Neutron scattering study of NH4- and acid proton “freezing” in Rb_1-x_(NH_4_)_x_H_2_PO_4_. Physica B 156 & 157 192-194 (1989).

[S6] Moussa, F. & Courtens, E. Pressure effect on the proton Rb_1-x_(NH_4_)_x_H_2_PO_4_: A neutron study. Ferroelecrrics 236, 181-192 (2000).

[S7] Trybula, Z., et al. Proton glass state in Rb_1-x_(NH_4_)_x_H_2_AsO_4_. Physica B 153, 143-146 (1988).

[S8] Trybula, Z., Stankowski, J. & Blinc, R. Proton glassy state of Rb_1-x_(NH_4_)_x_H_2_AsO_4_. Ferroelectrics Letters 6, 57-60 (1986).
